# Supplementary figures and images for: Trim41 is required to regulate chromosome axis protein dynamics and meiosis in male mice
Source: PLoS Genet. 2022 Jun 1;18(6):e1010241. doi: 10.1371/journal.pgen.1010241 (PMC9191731; doi:10.1371/journal.pgen.1010241)

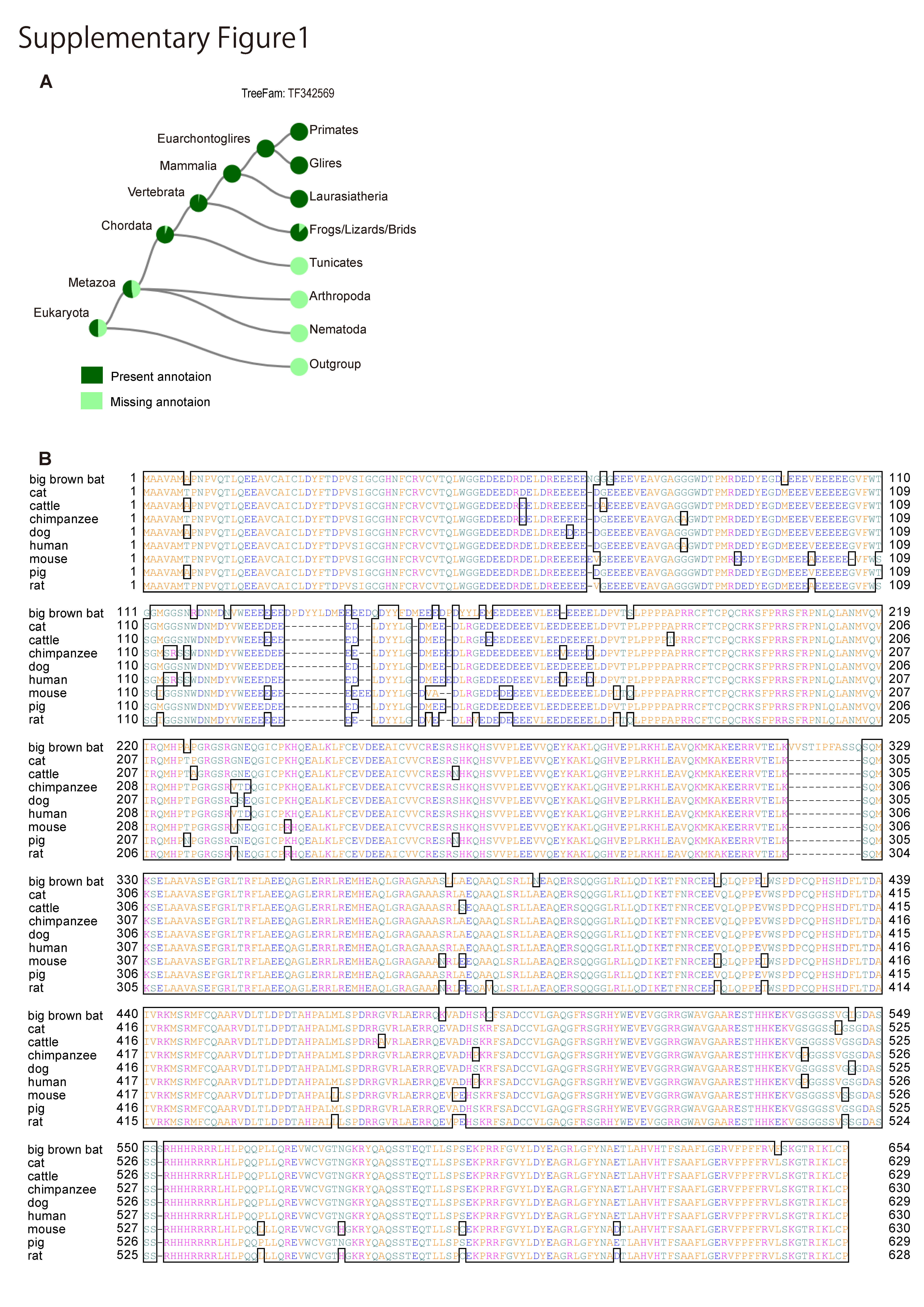

Supplement: S1 Fig — (A) The percentage of TF342569 annotated species based on the TreeFam database (Release 9; http://www.treefam.org/). Dark and light green show species with and without TF342569 annotation, respectively. (B) Protein sequence comparison of TRIM41 in big brown bat (XP_027991236.1), cat (XP_003980687.1), cattle (NP_001193094.1), chimpanzee (XP_016809993.1), dog (XP_038536985.1), human (NP_291027.3), mouse (NP_663352.2), pig (XP_020939058.1), and rat (NP_001128209.1). (TIF) [file pgen.1010241.s001.tif]

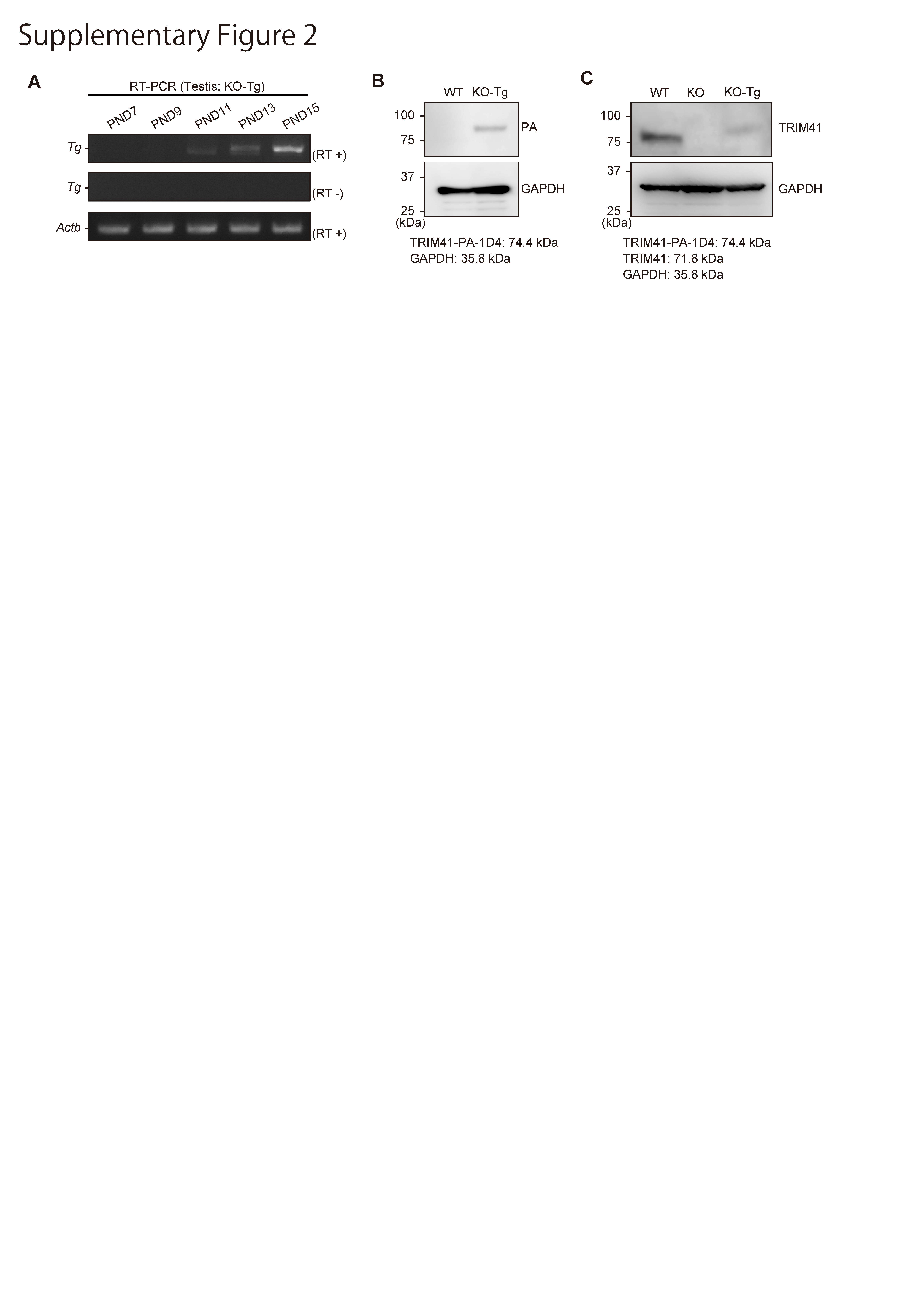

Supplement: S2 Fig — (A) RT-PCR using postnatal testis of Trim41 KO-Tg male mice. (B) Immunoblotting analysis with an anti-PA antibody. GAPDH was used as a loading control. (C) Immunoblotting analysis with an anti-TRIM41 antibody raised against recombinant TRIM41 (35–85 amino acid residues). GAPDH was used as a loading control. (TIF) [file pgen.1010241.s002.tif]

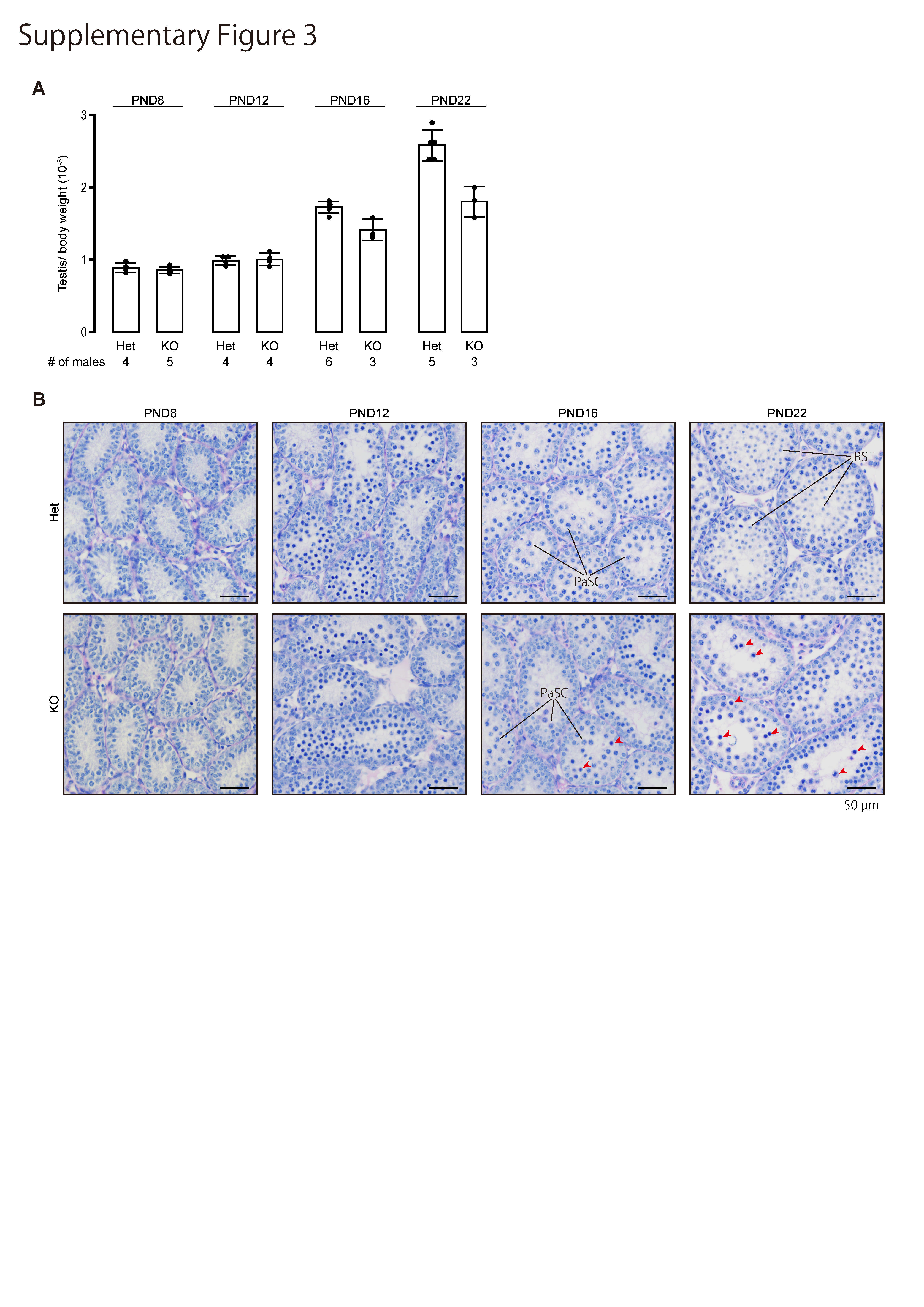

Supplement: S3 Fig — (A) Testis/bodyweight of Trim41 Het and Trim41 KO juvenile mice. PND8, PND12, PND16, and PND22 correspond to when the first wave of spermatogenesis reaches meiotic entry, zygotene stage, pachytene stage, and round spermatid occurrence, respectively. Testis/bodyweight: 0.89±0.07 x 10^−3^ [PND8-Het], 0.99±0.06 x 10^−3^ [PND12-Het], 1.72±0.08 x 10^−3^ [PND16-Het], 2.58±0.21 x 10^−3^ [PND22-Het], 0.86±0.05 x 10^−3^ [PND8-KO], 1.00±0.09 x 10^−3^ [PND12- KO], 1.41±0.15 x 10^−3^ [PND16-KO], 1.80±0.21 x 10^−3^ [PND22-KO]. Error bars indicate standard deviation. The numerical data are available in S3 Table (B) PAS staining of seminiferous tubules of juvenile mice. PaSC: pachytene stage spermatocyte; RST: round spermatid. Red arrowheads indicate apoptotic germ cells. (TIF) [file pgen.1010241.s003.tif]

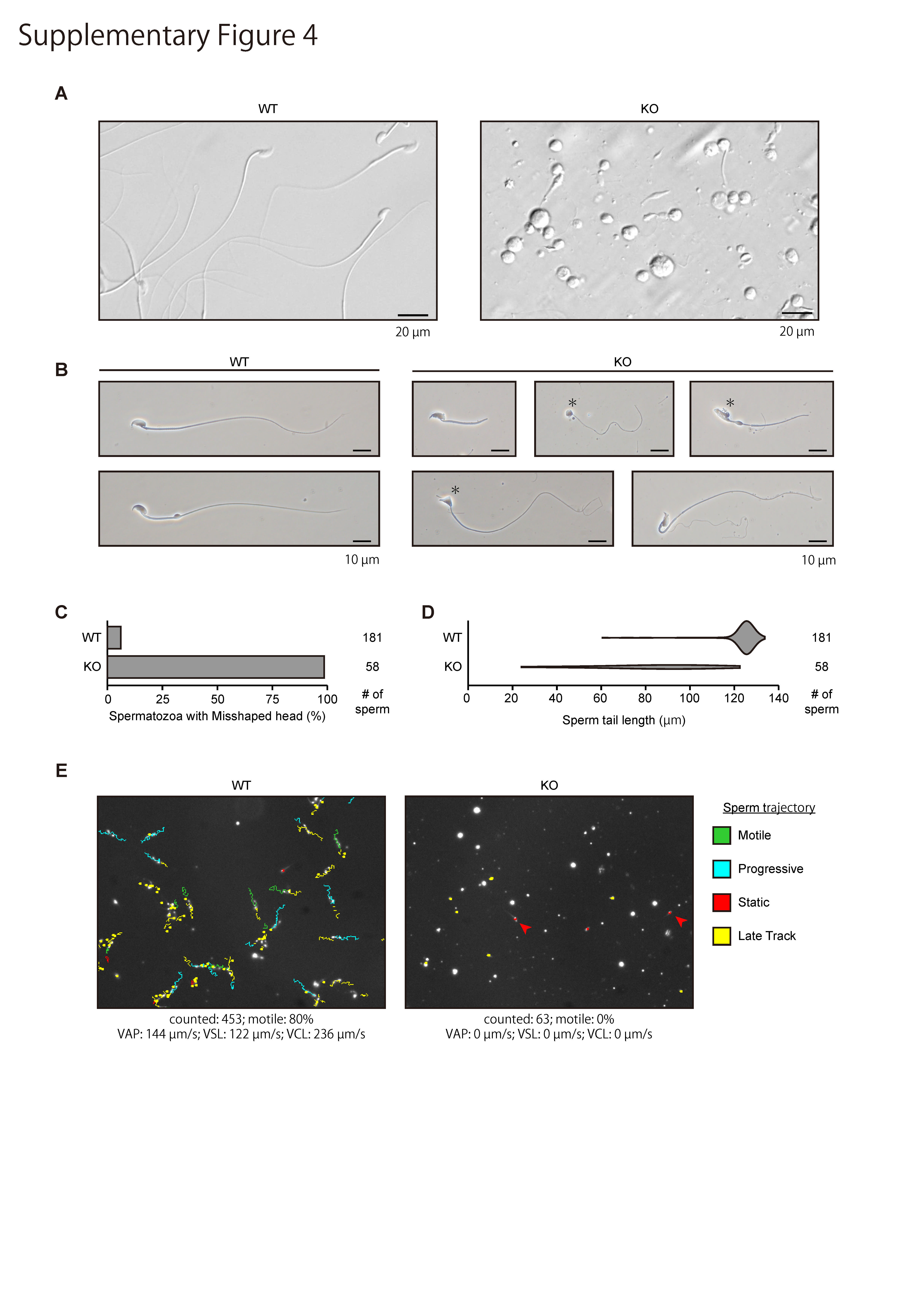

Supplement: S4 Fig — (A) Cells extracted from cauda epididymis were suspended in PBS containing 1% glutaraldehyde. The cell suspension on a plastic dish was observed by an inverted microscope. (B)The fixed cell suspension was dropped onto a glass slide, and a coverslip and gentle pressure were applied. The slides were observed with an upright phase-contrast microscope. Black asterisks indicate spermatozoa with misshaped heads. (C) The percentage of spermatozoa with misshaped heads: 11/181, 6% [WT]; 56/5, 97% [KO]. (D) A violin plot of sperm tail length: 124 ± 8 μm [WT], 86 ± 23 μm [KO] (s.d.). The numerical data are available in S3 Table (E) Motility analysis of cauda-extracted cells. The first frame of the analyzed movie and trajectory of cells are shown. Red arrowheads in the KO image indicate cells with a flagella(-like) structure. (TIF) [file pgen.1010241.s004.tif]

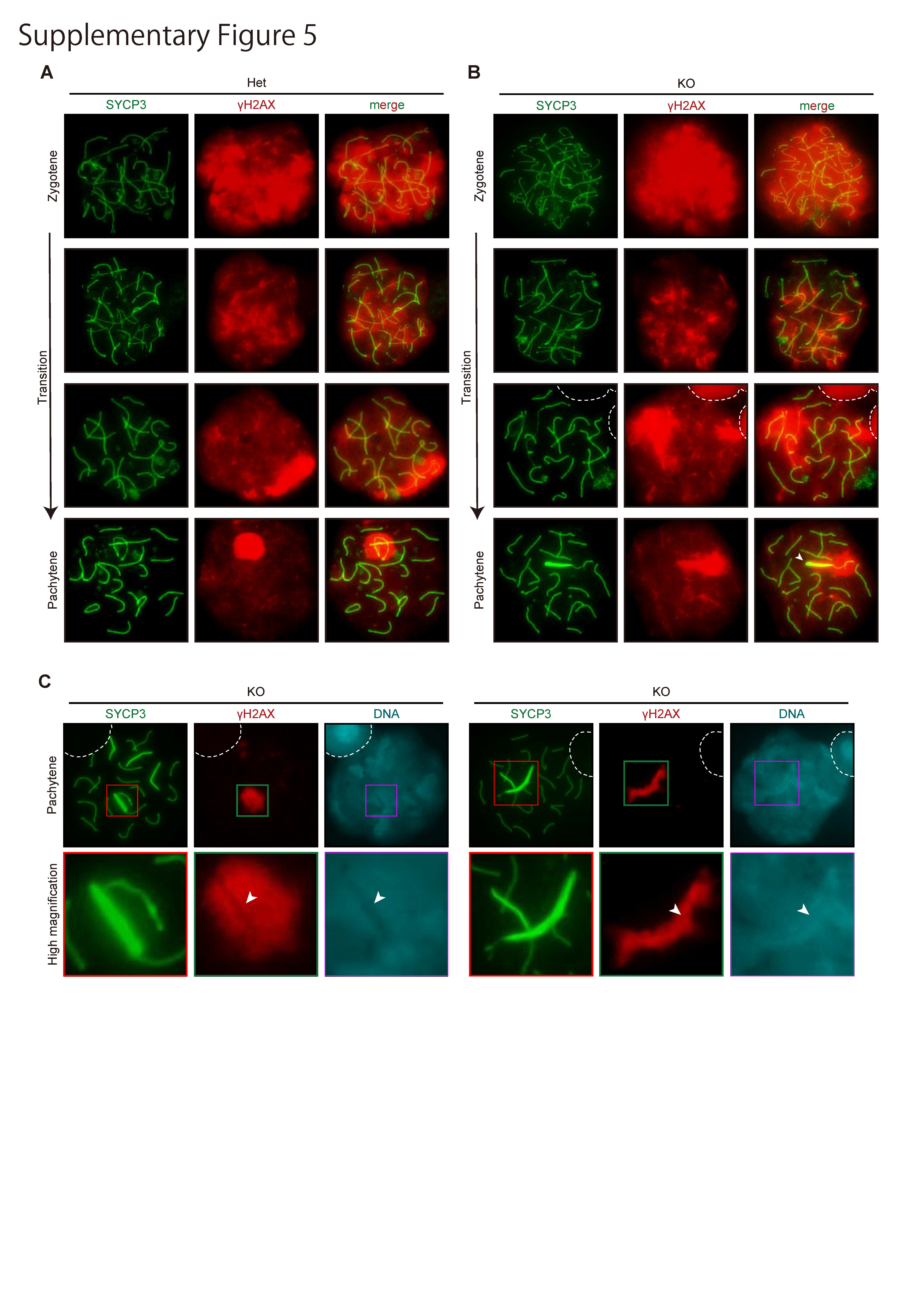

Supplement: S5 Fig — (A and B) Additional SYCP3/γH2AX immunostaining images of zygotene–pachytene stage spermatocytes from Het (A) and KO (B) male mice. The white dashed line shows nuclei of different cells. The white arrowhead indicates SYCP3 overloading. (C) KO pachytene stage spermatocytes stained with anti- SYCP3/γH2AX antibodies and DAPI. The white dashed line shows nuclei of different cells. White arrowheads indicate missing γH2AX/DNA signals. High magnified images are shown in red, green, and purple boxes. (TIF) [file pgen.1010241.s005.tif]

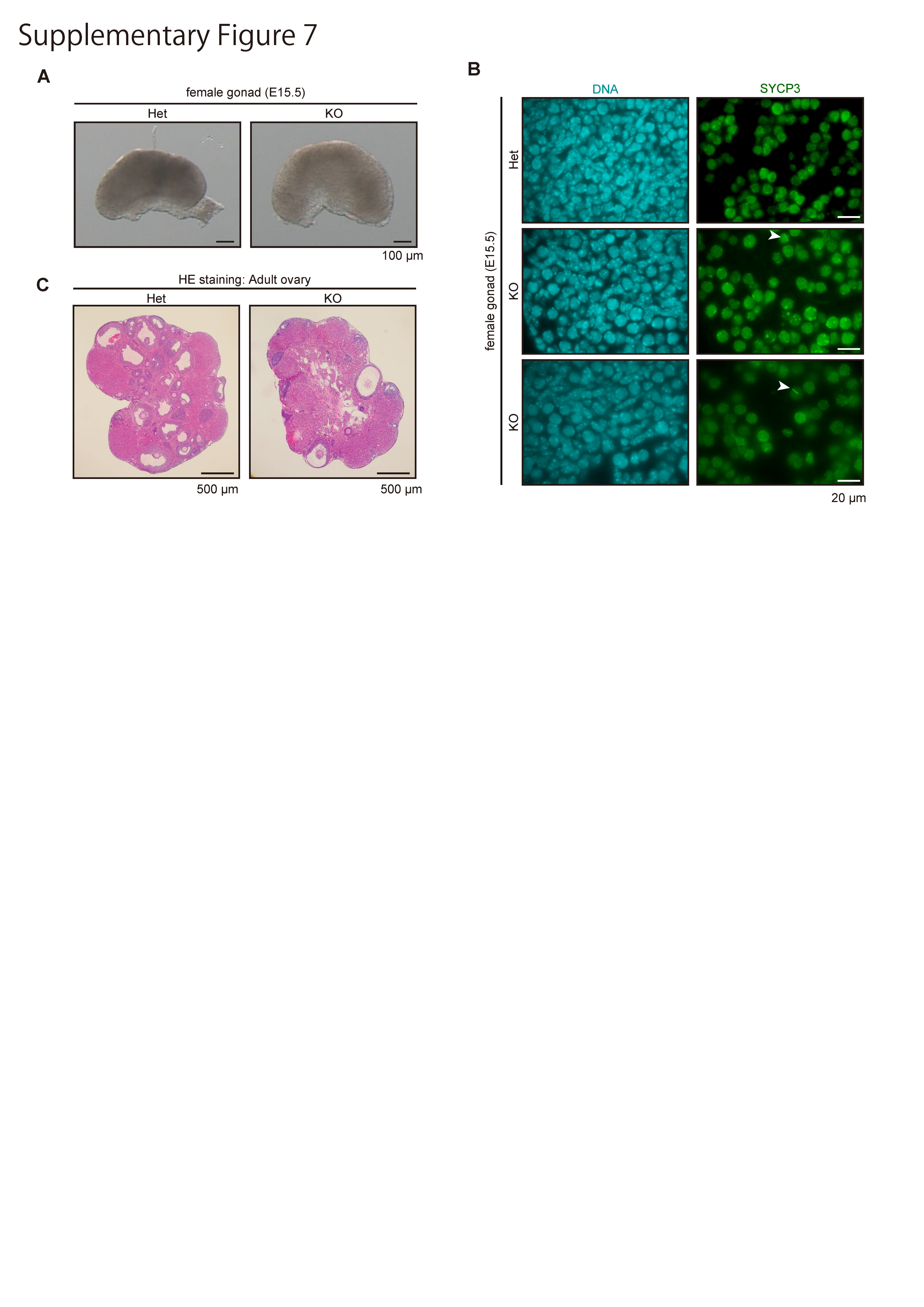

Supplement: S7 Fig — (A) Gross morphology of female gonads from E15.5 embryos. (B) Immunostaining of female gonad sections. White arrowheads indicate intense SYCP3 signals. (C) Ovarian histology of Trim41 Het and KO females. The ovaries were collected 10 h after intraperitoneal administration of hCG (58 h after PSMG administration). (TIF) [file pgen.1010241.s007.tif]

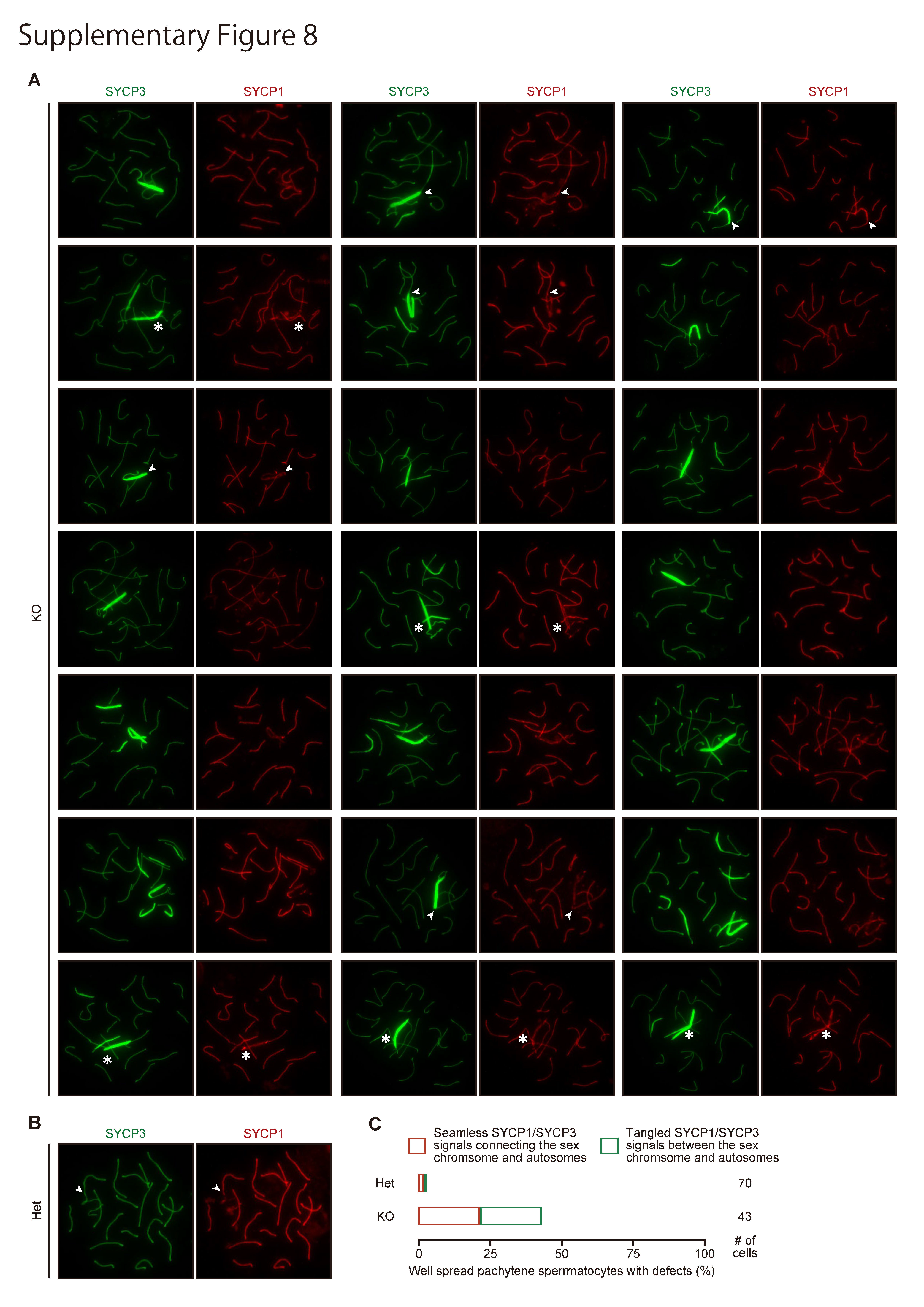

Supplement: S8 Fig — (A) Representative well-spread pachytene stage spermatocytes are shown (The first 18 pachytene spermatocytes were shown). White arrowheads show the seamless connection of SYCP1/SYCP3 signals. White asterisks indicate tangled SYCP1/SYCP3 signals. (B) An example of a Het pachytene stage spermatocyte with a seamless connection of SYCP1/SYCP3 signals. (C) Quantification data for SYCP1/SYCP3 staining. Red boxes indicate the percentage of pachytene spermatocytes exhibiting seamless connection of SYCP1/SYCP3 signals (1/70 [Het]; 9/43 [KO]). Green boxes show the percentage exhibiting tangled SYCP1/SYCP3 signals (0/70 [Het]; 9/43 [KO]). (TIF) [file pgen.1010241.s008.tif]

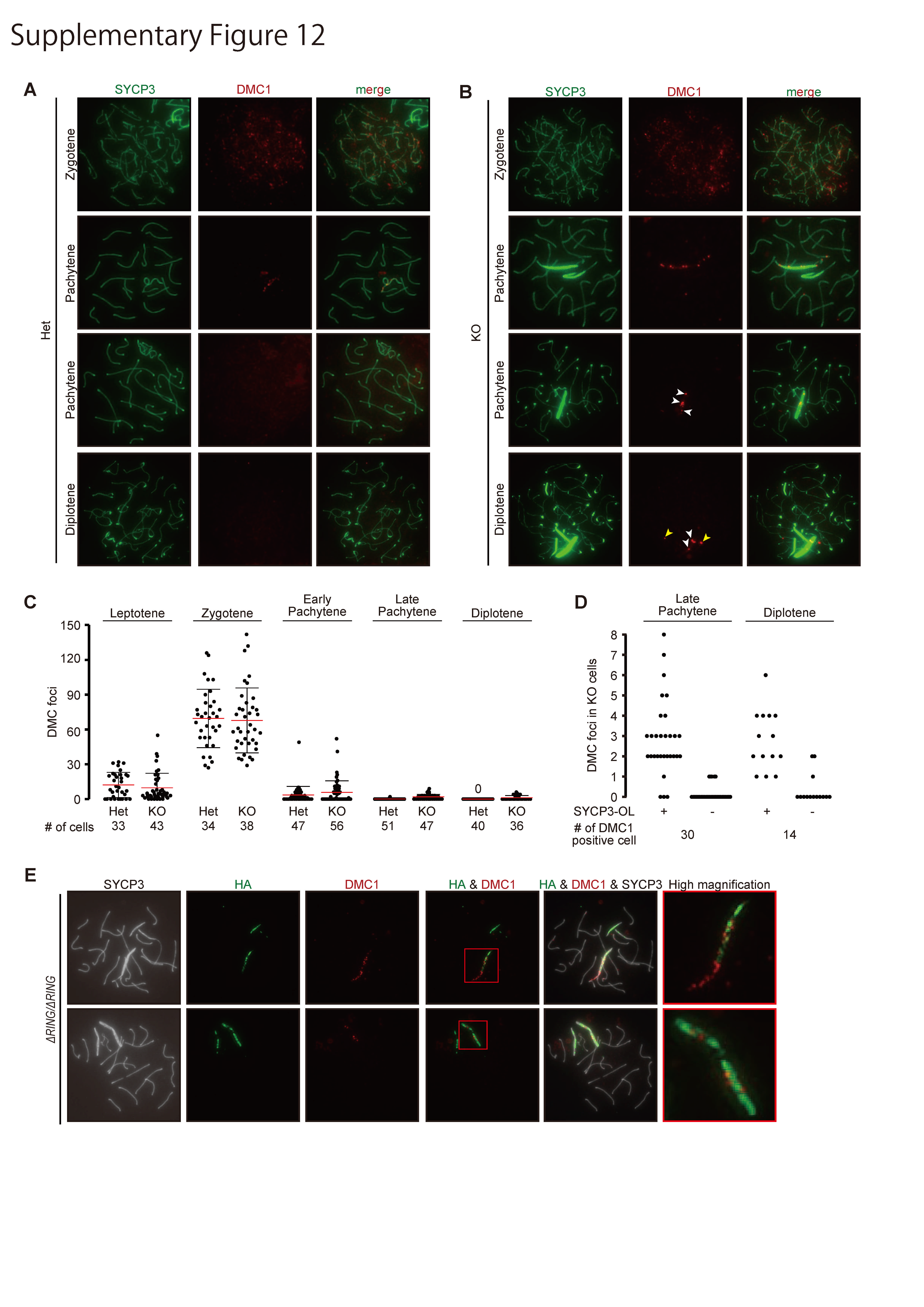

Supplement: S12 Fig — (A and B) The spread nuclei of prophase spermatocytes collected from adult Het (A) and KO (B) male mice were immunostained with anti-SYCP3 and -DMC1 antibodies. White and yellow arrowheads indicate remaining DMC1 foci on overloaded SYCP3 and normal-looking SYCP3, respectively. (C) The number of DMC1 foci is shown as a scatterplot with average and error bars (s.d.). Heterozygous diplotene stage spermatocytes had no DMC foci. (D) The number of DMC foci in KO late-pachytene and diplotene stage spermatocytes is shown as a scatterplot. Plus and minus in SYCP3-OL indicate DMC1 foci on overloaded SYCP3 and normal-looking SYCP3, respectively. (E) The spread nuclei of prophase spermatocytes collected from adult Trim41ΔRING/ΔRING male mice were immunostained with anti-SYCP3, -HA, and -DMC1 antibodies. High magnified images are shown in red boxes. (TIF) [file pgen.1010241.s012.tif]

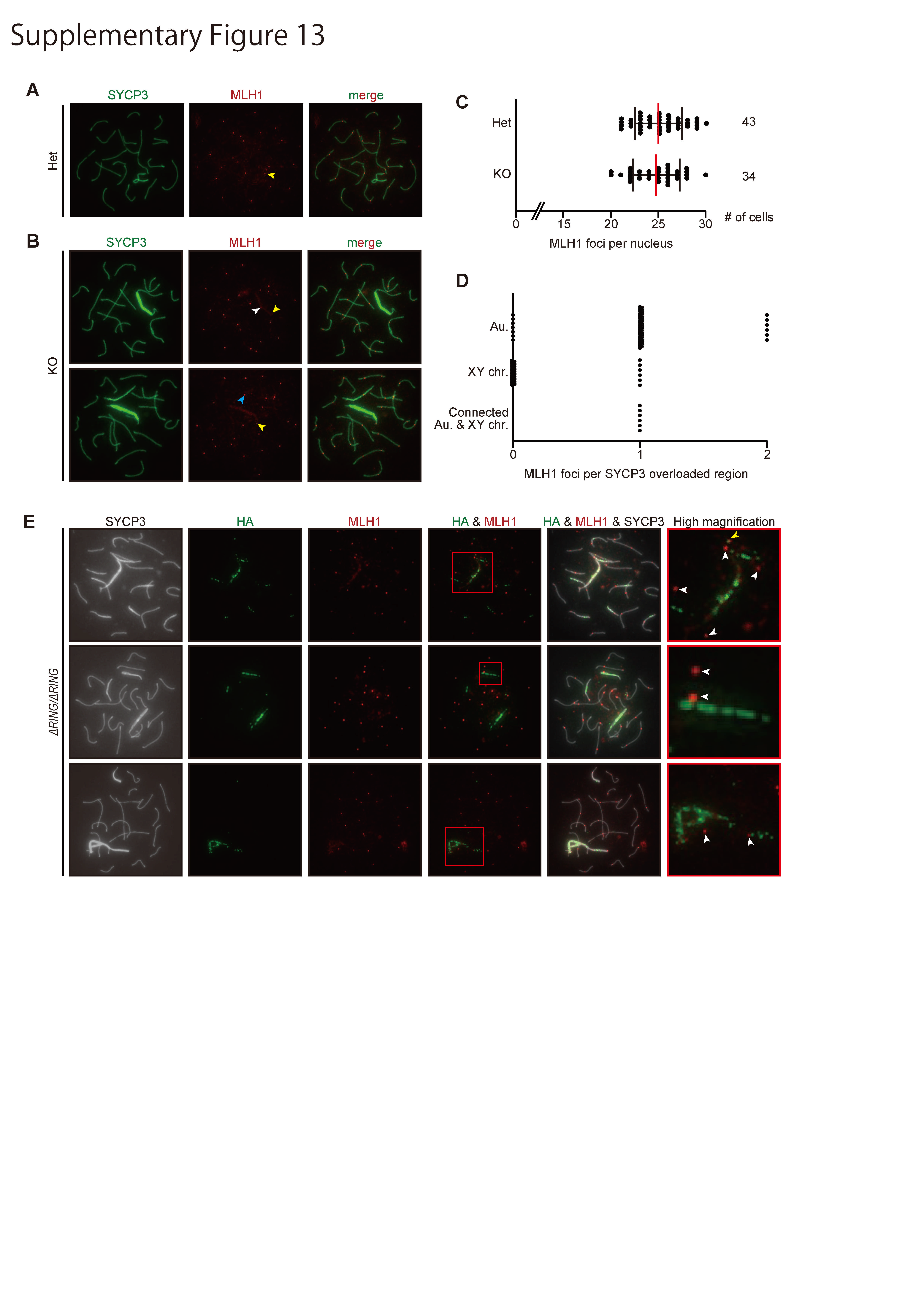

Supplement: S13 Fig — (A and B) The spread nuclei of prophase spermatocytes collected from adult Het (F) and KO (G) male mice were immunostained with anti-SYCP3 and -MLH1 antibodies. Yellow arrowheads indicate MLH1 foci on the pseudo-autosomal regions (PARs). The white arrowhead shows MLH1 foci on seamless SYCP3 signals connecting the sex chromosomes and autosomes. The blue arrowhead shows MLH1 foci on SYCP3-overloaded regions of autosome. (C) The number of MLH1 foci is shown as a scatterplot with average and error bars (s.d.). (D) The number of MLH1 foci on SYCP3-overloaded regions. (E) The spread nuclei of prophase spermatocytes collected from adult Trim41ΔRING/ΔRING male mice were immune-stained with anti-SYCP3, -HA, and -MLH1 antibodies. High magnified images are shown in red boxes. Yellow and white arrowheads in the highly magnified images indicate MLH1 foci colocalized with HA signals and not, respectively. (TIF) [file pgen.1010241.s013.tif]

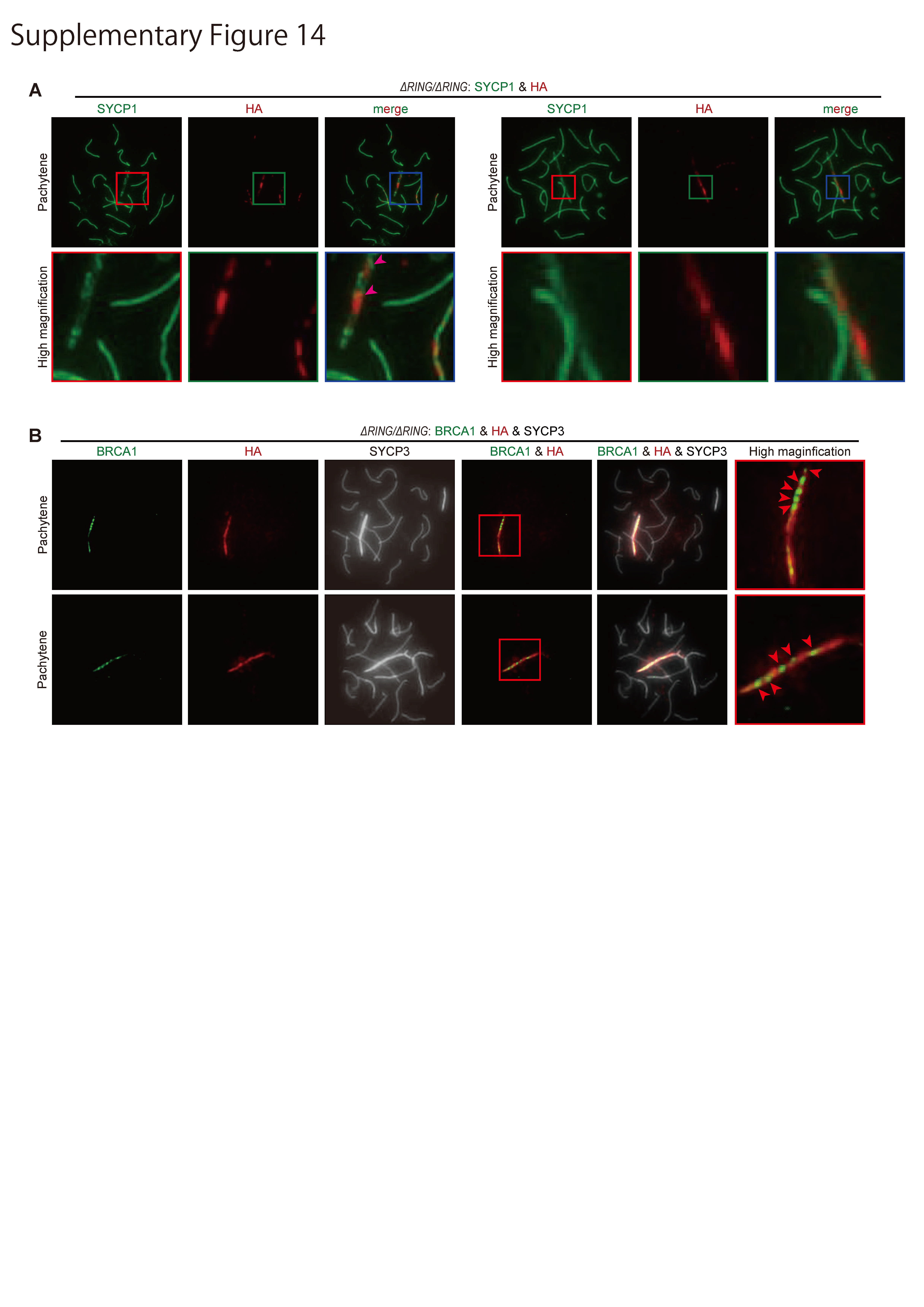

Supplement: S14 Fig — (A) SYCP1/HA immunostaining of surface chromosome spreads from Trim41ΔRING/ΔRING testis. Magenta arrowheads indicate HA signals on the SYCP1 positive/negative boundary. High magnified images are shown in red, green, and blue boxes. At least three male mice were analyzed. (B) BRCA1/HA immunostaining of surface chromosome spreads from Trim41ΔRING/ΔRING testis. Red arrowheads indicate HA signals on the BRCA1 negative part of the X chromosome axes. High magnified images are shown in red boxes. At least three male mice were analyzed. (TIF) [file pgen.1010241.s014.tif]

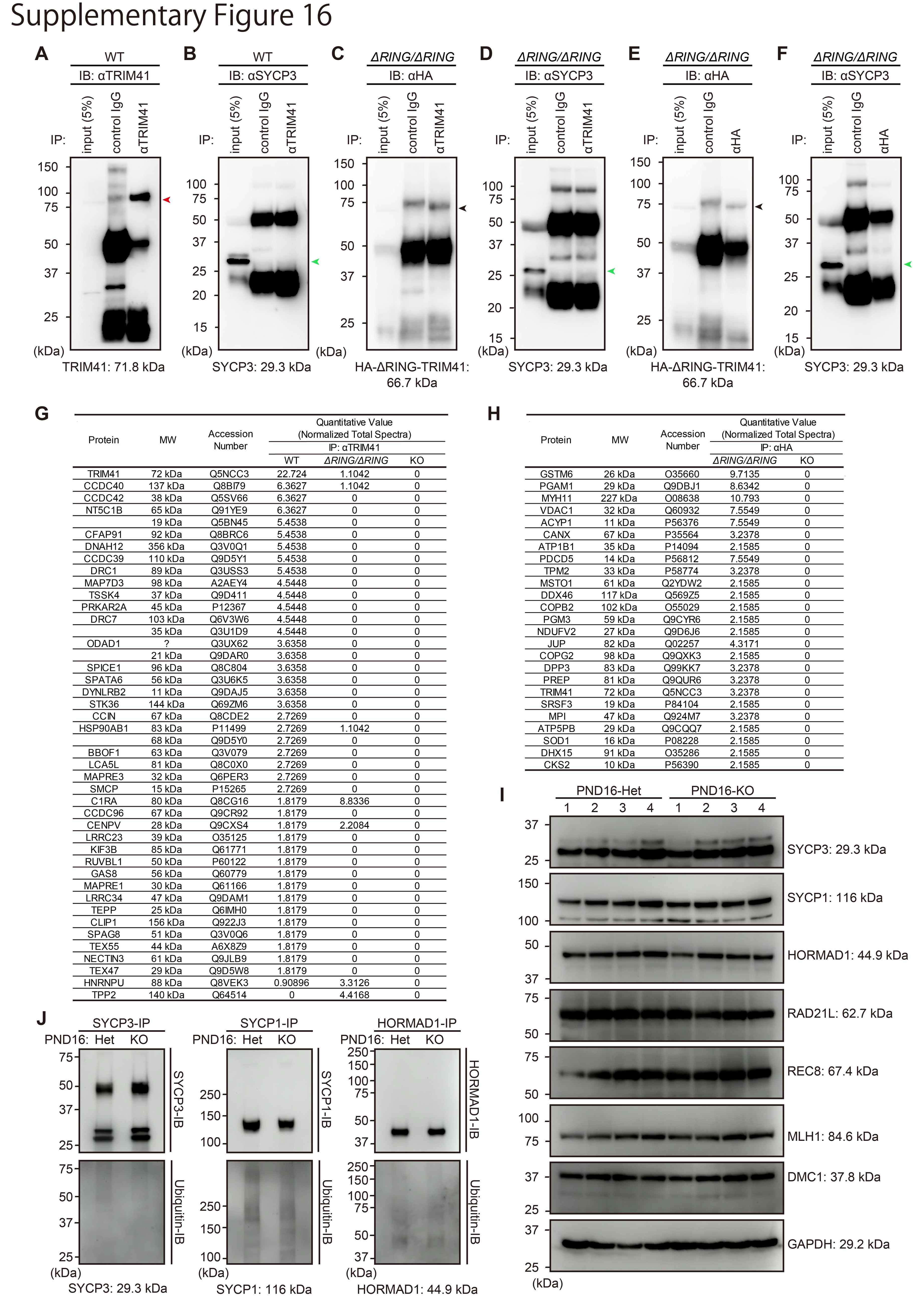

Supplement: S16 Fig — (A and B) Immunoprecipitation using an anti-TRIM41 antibody and WT testis lysate. TRIM41 immunoblotting (A) validated the immunoprecipitation capability of the anti-TRIM41 antibody and the success of immunoprecipitation experiments. Red and green arrowheads indicate TRIM41 and SYCP3, respectively. (C and D) Immunoprecipitation using an anti-TRIM41 antibody and Trim41ΔRING/ΔRING testis lysate. HA immunoblotting (C) validated the success of immunoprecipitation experiments. Black and green arrowheads indicate ΔRING-TRIM41 and SYCP3, respectively. (E and F) Immunoprecipitation using an anti-HA antibody and Trim41ΔRING/ΔRING testis lysate. HA immunoblotting (E) validated the success of immunoprecipitation experiments. Black and green arrowheads indicate HA-ΔRING-TRIM41 and SYCP3, respectively. (G and H) Mass analysis of co-IPed eluates using anti-TRIM41 (G) and anti-HA (H) antibodies. Proteins with 0 spectra in KO lysate are extracted and summarized in the table. The whole proteomics data are available in S4 Table (I) Immunoblotting of chromosome axis proteins using PND16 testis lysates. 4 Het and KO littermates were examined. PND16 testis was used to minimize the effect of cell population differences. (J) Immunoprecipitation of chromosome axis proteins, followed by immunoblotting using an anti-Ubiquitin antibody. PND16 testicular germ cells were lysed in NP40 lysis buffer [50 mM Tris-HCl (pH 7.5), 150 mM NaCl, 0.5% NP-40, 10% Glycerol] supplemented with a cocktail of protease inhibitor and a DUB inhibitor (20 μM PR619). (TIF) [file pgen.1010241.s016.tif]
