# Supplementary material for: Trim41 is required to regulate chromosome axis protein dynamics and meiosis in male mice
Source: PLoS Genet. 2022 Jun 1;18(6):e1010241. doi: 10.1371/journal.pgen.1010241 (PMC9191731; doi:10.1371/journal.pgen.1010241)
Supplement: Attachment — Submitted filename: Reponse to reviewers.docx [file pgen.1010241.s022.docx]

**Manuscript number:** RC-2021-01140

**Corresponding author:** Masahito, Ikawa

**General comments:**

We appreciate the reviewers for their valuable comments. We realized that statements on self or non-homologous synapsis were too much to say. We removed these phrases from the title, introduction, and result sections, and mentioned the idea as a hypothesis in the discussion section. In this manuscript, we focus on the phenotypical description of *Trim41* KO spermatocytes, and the exact role of TRIM41 and abnormal synapsis will be the future consideration.

**Response to the reviewers:**

**Reviewer #1 (Evidence, reproducibility and clarity (Required)):**

In the manuscript, Ikawa and co-authors provide evidence of a novel and unexpected role for an E3 ubiquitin ligase, TRIM41, in meiosis in male mice. The initial characterization of the meiocytes of mutant mice (or mice lacking the conserved RING domain of TRIM41) indicates a problem in loading of a component of the synaptonemal complex, SYCP3, concomitant with aberrant synapsis. Likely as a consequence, apoptosis levels are up, and the mice are sterile.

The manuscript provides an initial, descriptive characterization of the role TRIM41 plays in mice meiosis, along with some tantalizing ideas about what might be the exact role TRIM41 plays. Future research to define the mechanism of TRIM41 action is bound to be exciting. While some of the specific claims are imprecise or overstated and some quantitations are missing, the data is overall of high quality, and the impact of TRIM41 on male meiosis in mice is well substantiated.

Due to my area of expertise, I will refrain from commenting on specific experimental details concerning analysis of meiosis in mice. I will limit my comments to more universal features of meiotic chromosome dynamics, and to the logic and clarity of the manuscript.

**Major comments:**

In Fig. 4, the mis-localization of SYCP1 is apparent. However, some quantitation of SYCP1 and SYCP3 on unaffected chromosomes (i.e., not on the bright rods of SYCP3) would be helpful. Is SYCP3 depleted on other chromosomes? Is SYCP1? For example, in the images shown for pachytene in Fig. 3A-B, it seems like the telomeric enrichment of SYCP3 relative to SYCP1 seen in the Het is less prominent in the KO. Is this true?

- We reanalyzed randomly taken images and did not observe depletion or other apparent defects in SYCP1 and SYCP3 on other chromosomes. We mentioned this point in the manuscript (L.202-203, L. 261-263).

L.202-203: “We did not observe apparent abnormalities other than in SYCP3-overloaded regions.”

L.261-263: “These defects in SYCP1, BRCA1, and HORMAD1 localization patterns were observed only in SYCP3-overladed regions.”

- The different telomeric enrichment of SYCP3 between Het and KO was due to the stage mismatch of pictures which we showed in Fig. 3A-B. We changed the photo to avoid misleading the readers.

The bright, rod-like, SYCP3 structures are one of the striking features of the TRIM41 KO mice. However, given the current state of their characterization, some of the statements regarding them should be more carefully phrased. The term 'polycomplex-like' (e.g., line 217) should probably be avoided; polycomplexes are not associated with chromatin and have repetitive striations reminiscent of SC assembled onto chromosomes. The first part is not true in this case, and the second part has not been addressed in this work. Similarly, the claims regrading 'multilayer axes' (line 202) or multiple chromosomes synapsing (e.g., line 266-267) are not currently supported by the data. The authors provide evidence that the X chromosome is mostly associated with the rod-like structure in metaphase and in pachytene; but in order to make any claims as to the nature or existence of multi-chromosome associations, more analysis would be necessary (e.g., FISH on pachytene spreads).

- We thank the reviewer for pointing out of this. We noticed these terms and phrases were exaggerated and deleted “multilayer axes” and “polycomplex-like” from the result section.
- Although we tried to capture multi-chromosome associations by multicolor FISH on pachytene spreads, we could not distinguish multi-chromosome associations and proximal placement (i.e., synapsed or not). We have decided to avoid mentioning abnormal synapsis, as noted above.

The claims as to the specificity of the phenotype to male meiosis should also be soften. The authors clearly demonstrate that only male trim41 KO (and ΔRING) mice exhibit sterility and strong meiotic phenotypes. However, the claim in lines 241-242 suggests that the reason for the male-specific phenotypes has to do with the asynapsed X. That is an interesting hypothesis, but it should be tested. Based on the evidence currently presented, it is possible that either it is the presence of asynapsis OR the physiology of male meiosis that underlies the different effects on males and females.

- We again thank the reviewer for pointing out the overstatement. As the reviewer mentioned, additional tests are needed for the “asynapsed” hypothesis, such as *Trim41*-deficient XO meiotic progression. Although this is an intriguing analysis, we have decided not to carry out this because the study is beyond the scope of this revision.
- We removed the hypothesis from the result section.

In order to make this manuscript more accessible, some of the techniques and antibodies used should be better explained. Specifically, the legend for Figure 3 is quite unclear, as is its explanation in the text.

- We listed all antibodies used in this study in supplemental table S2. In addition, throughout the manuscript, we revised the sentences for a better and more detailed explanation of figures (especially Fig. 3).

There are quite a few language errors, as well as imprecisions regarding the meiotic process and general missing explanations. Several examples:

- We asked for English proofreading and revised language errors.

Line 67: The use of the word 'showed' is premature; the data barely 'suggests' this finding. It really is just a speculation at this point.

- We appreciate the reviewer for the clinical comment. We changed the word “showed” into “suggested”.

Lines 91-92: The beginning of that sentence does not make sense. Similarly, the first sentence of the abstract is incorrect: meiosis does not 'accompany sequential chromosome events'.

- We removed the word “chromosome” from the abstract sentence and redundant information from the introduction sentence.

Line 244: the word 'saw' is inappropriate. Do the authors mean 'sow'? Or 'cause'?

- We changed the subtitle.

Lines 201-210: SYCP3 is mis-spelled multiple times.

- We revised all the mistakes.

Lines 258-267: the role of BRCA1 and the mechanism of MUSC should be better explained.

- Considering other reviewers’ comments, we removed the sentences regarding MUSC.

Line 283: 'as well as' is used incorrectly.

- We revised the mistake.

Lines 285-291: Understanding this section requires prior knowledge of the organization of the mammalian testis and the role of apoptosis in meiosis. These should be better described to make the manuscript accessible to a broader audience.

- As we did the same experiment in Fig.2, we added an explanation of germ cell composition in mouse testis in L.156-164.

L.156-164: “To define the cause of testicular atrophy, we performed hematoxylin and periodic acid-Schiff (HePAS) staining of testicular sections and found that the germ cell layer was thinner in Trim41 KO testis than in control testis (Fig. 2C; low magnification). Then we compared testicular cells based on the cycle of the seminiferous epithelium [21, 22]. From this analysis, we determined the population of the first germ cell layer containing pleleptotene stage (Fig. 2C; stage VII–VIII) and zygotene stage (Fig. 2C; stage XII) was comparable between Het and KO testis. On the other hand, pachytene stage (Fig. 2C; stage VII–VIII) and diplotene stage (Fig. 2C; stage XII) spermatocytes and spermatids were dramatically decreased in the KO testis.”

- We added two references regarding meiotic checkpoints and the phrase “failed in meiotic processes” (L.172-173).

L.172-173: “These results suggested that spermatocytes after the pachytene stage might failed in meiotic processes [23, 24].”

[23] Hochwagen A, Amon A. Checking your breaks: surveillance mechanisms of meiotic recombination. Current biology : CB. 2006;16(6):R217-28.

[24] Subramanian VV, Hochwagen A. The meiotic checkpoint network: step-by-step through meiotic prophase. Cold Spring Harbor perspectives in biology. 2014;6(10):a016675.

The data in Figure 1E-F should be better explained and contextualized. In their current form these panels add little to the figure.

- We added the sentence describing Figure 1E-F.
- Considering other reviewers’ comments, we moved Fig.S1B-C to the main figure.

In Fig. 5B, F1-R1 primers are probably mislabeled.

- We revised the mistake.

Reviewer #1 (Significance (Required)):

As noted in the general comments, due to my area of expertise, I am limiting my comments to universal features of meiotic chromosome dynamics, and to the logic and clarity of the manuscript.

The results of this paper are novel and surprising. They will be of interest to anyone studying meiotic chromosome dynamics, and particularly those who study the synaptonemal complex or mice meiosis. However, in its current form, the manuscript is descriptive and adds little to our understanding of the meiotic program beyond implicating TRIM41 in it. The most exciting aspects of the work are currently at the level of preliminary and suggestive observations that will require substantiation in order to be meaningful.

Several suggestions for ways to substantiate some of these findings:

The authors document a defect of crossover formation that accompanies the synapsis defect. It would be interesting to observe earlier markers of crossover formation in pachytene (MSH5, MLH1, etc.) in order to document how their loading is affected by TRIM41 elimination, and whether they have a particular association pattern with the bright rod-like SYCP3 structures.

- We thank the reviewer for the suggestion. We quantified MLH1 foci and incorporated the data into the manuscript (Figs S13 and L.328-333). Briefly, overall MLH1 loading activity was not affected by TRIM41 disruption. Also, we could not find a particular association pattern of MLH1 with the bright rod-like SYCP3 structures.

L.328-333: “We next examined the MLH1 foci, a DNA-mismatch protein promoting cross-over during meiosis [36, 37]. The number of MLH1 foci per nucleus was comparable between Het and KO spermatocytes (Figs S13A–S13C). We did not observe either the specific association pattern between MLH1 foci and overloaded SYCP3 (Fig. S13D) or colocalization of MLH1 foci and ΔRING-TRIM41 (Fig. S13E). These results suggested that TRIM41 did not directly regulate recombination protein dynamics.”

While the authors didn't manage to find direct interaction between SYCP3 and TRIM4, a tantalizing mechanism is that TRIM41 regulates the levels of SYCP3. One way to test this idea is to quantify the amount of SYCP3 in wildtype and KO mice.

- We performed immunoblotting to examine the amount of SYCP3 (and other proteins localizing on chromosome axes: SYCP1, HORMAD1, RAD21L, REC8, MLH1, DMC1) using PND16 testis to minimize the effect of cell population difference. However, unfortunately, we could not find apparent protein level deference between Het and KO. We described this result in Fig S16I and L. 359-361.

L.359-361: “The protein amount and ubiquitination level of several axis-related proteins, including SYCP3, SYCP1, HORMAD1, were comparable between Het and KO testis (Fig. S16I and S16J).”

**Reviewer #2 (Evidence, reproducibility and clarity (Required)):**

In this paper by Oura et al, the authors analysed meiotic phenotypes of Trim41 knockout (KO) male mice. TRIM41 is a E3 ubiquitin ligase and its meiotic roles have been elusive. The authors found that Trim41 KO male mice were infertile and showed defects in meiosis. Trim41 KO spermatocytes exhibited SYCP3 overloading as well as defects in homologous synapsis. The deletion of the RING domain (ΔR) phenocopied the KO mice suggesting that the E3 ubiquitin ligase activity is likely required for the meiotic functions. ΔR-TRIM41 was observed accumulating on the chromosome axes with overloaded SYCP3, suggesting that TRIM41 may function on the chromosome axes.

The work used three mouse models, KO, transgenic, and ΔR and performed comprehensive phenotypic analyses. The paper will be high quality one if continuing to study the detailed mechanism for why the SYCP3 overloading happens after Trim41 disruption. Unfortunately, at this point, the paper solely focuses on the phenotypic description, lacks any solid support of the possible mechanism, and will not be recommend for publication right now.

Major point

1, TRIM41 is a ubiquitin ligase and most likely is involved in ubiquitination of chromosomal axis proteins. However, the author did not provide any mechanical insight on this. A possible experiment is the immunoprecipitation of chromosome axis proteins, such as SYCP3, SYCP2, REC8, and RAD21L, from WT and KO testes followed by blotting with ubiquitin antibodies, which could tell the difference in the axis-associated ubiquitin levels between WT and KO. Alternatively, the western blotting of WT and KO chromatin extracts with the ubiquitin antibody could tell the difference.

2, Another possible experiment is a more comprehensive proteomics analysis targeting for the ubiquitinated proteins (for example; mass spectrometry analysis after ubiquitin IP from WT and KO testis extracts).

- We thank the suggestion. We tried immunoprecipitation of several chromosome axis proteins with abnormal staining patterns in KO (i.e. SYCP1, SYCP3, HORMAD1), followed by immunoblotting with ubiquitin antibodies. However, we could not find apparent ubiquitin level deference between Het and KO. We described this result in Fig S16J and L. 359-361.

L.359-361: “The protein amount and ubiquitination level of several axis-related proteins, including SYCP3, SYCP1, HORMAD1, were comparable between Het and KO testis (Fig. S16I and S16J).”

3, In preceding studies, it is shown that ubiquitin localizes along chromosome axes in spermatocytes (PMID: 28059716). Ubiquitin staining in the WT and KO should be provided.

- We stained ubiquitin in the spread nuclei (Figs S15). However, we did not observe ubiquitin-loss in KO spermatocytes. On the other hand, the intensity of ubiquitin staining became stronger on SYCP3 overloaded axes, demonstrating that TRIM41-disruption did not affect overall ubiquitination on chromosome axes. We described this result in L. 352-356.

L. 352-356: “Given that TRIM41 confers ubiquitin ligase E3 activity [9, 10], we examined ubiquitin localization along chromosome axes [38]. However, the ubiquitin localization pattern was comparable between Het and KO spermatocytes (Figs S15A and S15B), showing the overall ubiquitination activity on chromosome axes was not affected by TRIM41 disruption.”

4, The localization of HA-TRIM41-ΔR appeared as a dotty signal along chromosome axis. The pattern is similar to the distribution of recombination nodules. Co-staining of HA with recombination markers such as RPA, RAD51, DMC1, and MSH4 should be provided.

- Thank you for the valuable suggestion. We examined the relationship with the distribution of recombination nodules by co-immunostaining of HA with DMC1 and MLH1 (Figs S12E and S13E, L.325-327, L.330-333). However, DMC1 and MLH1 did not colocalize with HA-TRIM41-ΔR. These results suggested that TRIM41 did not directly regulate recombination protein dynamics.

L.325-327: “However, DMC1 foci did not colocalize with HA-ΔRING-TRIM41, suggesting that the remaining DMC1 might be a downstream defect.”

L.330-333: “We did not observe either the specific association pattern between MLH1 foci and overloaded SYCP3 (Fig. S13D) or colocalization of MLH1 foci and ΔRING-TRIM41 (Fig. S13E). These results suggested that TRIM41 did not directly regulate recombination protein dynamics.”

Minor point

1, Figure 1E and 1F are not discussed in manuscript, and provide rather supplementary data.

2, From the Figure1, the authors concluded that TRIM41 was highly expressed in pachytene stage. The RT-PCR results in Figure 1E were not so clear to conclude the stage-specific mRNA level. The supplementary Figures 1 C and D seem to be more convincing and should be moved to the main figure.

- We described Figure 1E and 1F in more detail and moved the supplemental data to the main figure (Figure 1).

3, Figure 5C and 5D shows western blot data without proper loading controls.

- We added loading controls throughout the manuscript.

4, What the arrowheads in the immunostaining pictures indicate should be described not only in the main text but also in the individual figure legends.

- We added the explanation in each figure legend.

5, Figure 2G showed that there were abnormal head/tail shapes in Trim41 KO spermatozoa. However, the picture is not of high quality. Also there is no WT control. The author should provide more careful observation of the sperm morphologies with high quality images, controls, and quantifications.

- We changed the figure with a high-quality image and added a control image. Further, we quantified the abnormal morphology phenotype of spermatozoa and analyzed their motility (Figs S4A-4E, L. 178-179).

L.1778-179: “In addition, all the spermatozoa exhibited abnormal head/tail shapes (Figs S4A-4D) and were immotile (Fig. S4E).”

6, From the figure 6H, it will be hard to tell if it was pachynema or not without any stage markers, like SYCP3. Please add more staining to avoid misunderstanding if it is possible.

- We thank the reviewer for pointing this out. We obtained guinea pig-raised anti-SYCP3 antibody (Ishiguro et al., Dev Cell. 2020) and changed those figures with triple-stained (SYCP3/HA/BRCA1) spermatocyte images.

Reviewer #2 (Significance (Required)):

The paper will be of significant importance if continuing to study the detailed mechanism for why the SYCP3 overloading happens after Trim41 disruption. Unfortunately, at this point, the paper solely focuses on the phenotypic description, lacks any solid support of the possible mechanism.

**Reviewer #3 (Evidence, reproducibility and clarity (Required)):**

Summary:

In this manuscript Ikawa et al report that TRIM41 is an essential factor for proper meiotic progression and fertility in male mice. They develop 2 mice model, a mouse null for TRIM41 generated by gene targeting, and another mouse lacking the RING domain of TRIM41 (required for the ubiquitin ligase E3 activity) generated by genome editing. These both mutants mice are infertile and their spermatocytes exhibit SYCP3 overloading in pachytene chromosomes and especially on the X chromosome. They also show that ΔRING-TRIM41 accumulate on the chromosome axes overlapping with SYCP3. With these observations the authors conclude that Trim41 is essential for preventing SYCP3 overloading and chaotic synapsis of the X chromosome, and suggest a TRIM41-mediated mechanism for regulating unsyapsed axes during male meiotic progression.

Major Comments

The manuscript is merely descriptive and does not provide any mechanism to support their conclusions. The authors suggest that as ΔRING-TRIM41 accumulates on chromosomal axes with overloaded SYCP3 and on the synapsed part of the X chromosome axes. Thus they suggest that TRIM41 may act by removing overloaded SYCP3 and for preventing the chaotic X chromosome synapsis. However they were unable to co-immunoprecipitate SYCP3 with TRIM41 or provide any direct molecular evidence of a direct interplay between them. Moreover, as they themselves affirm, they have not been able to find any candidate protein that is a substrate for this ubiquitin ligase E3.

On the other hand, all the observations described have to be quantified and no conclusion can be reached before this quantification had been carried out . For example, the authors state that compaction of the XY axes was rarely observed in the KO. What does "rarely" means? What percentage of spermatocytes present an aberrant sex-body? Regarding Figure 3 and 4, how many spermatocytes show the X chromosome with multi-layered SYCP3 signal or with self or non-homologous synapsis?

- We added the quantification data of sex body malformation and abnormal loading of SYCP3 in randomly captured images (Fig. 3D and L. 192, 195-196)

Pachytene spermatocytes exhibiting XY body malformation: 6/181 [Het]; 192/207 [KO]

Pachytene spermatocytes with intense SYCP3 signals: 3/181 [Het]; 205/207 [KO]

- We added more SYCP1/SYCP3-stained images (Figs. S8A-C and L. 250-252) and the quantification data of tangled SYCP1/SYCP3 signals, which suggested non-homologous synapsis.

Seamlessly connected SYCP1/SYCP3 signals: 1/70 [Het]; 9/41 [KO]

Tangled SYCP1/SYCP3 signals: 0/70 [Het]; 9/41 [KO]

Figure S4D does not show clearly the dissociation of RAD21L in early pachytene as the authors claim. In this regard, they should look at MI to see if the overload of SYCP3 corresponds with Rec8 accumulation as in Pachytene stages or if cohesins are correctly eliminated.

- We thank the reviewer for pointing out this. We stained the spread nuclei again with the same anti-RAD21L and REC8 antibodies. However, the previously observed (n=3) spread cohesin signals became not observed both in Het and KO (again, n=3). The spread signals might be due to decreased quality of the long-stored previous batch of antibodies.
- We changed Figure S4 (Figure S5 in the revised manuscript) with newly stained images because the newly stained images were more similar to the ones in previous research using the same antibody (Genes Dev. 2014 Mar 15;28(6):594-607).

Reviewer #3 (Significance (Required)):

The presented data in this MS are based on shallow observations, and particularly their interpretation and presentation should be substantially improved before considering for publicaction. Consequently, the conclusions are not convincingly supported by the experimental data.

My expertise: Meiosis, fertility, chromosome segregation

**Reviewer #4 (Evidence, reproducibility and clarity (Required)):**

Summary:

In this manuscript, the authors provide an informative description of the male infertility phenotype of mice lacking Trim41, a ubiquitin ligase E3 protein. Although females with the KO mutation are fertile, the males are infertile, with an apparent block of spermatogenesis in the meiotic stage (more on this below). Surface-spread chromatin preparations coupled with IF revealed an "overloading" of the synaptonemal complex protein SYCP3, especially on the unsynapsed X chromosome, where it forms a crystalline-like aggregate. Mice lacking only the RING domain of TRIM41 phenocopied the KO, providing evidence that the ubiquitin ligase E3 activity is essential for normal accumulation of SYCP3 and X-chromosome dynamics. The information in the manuscript is clearly presented (although there is great need for English editing prior to publication). The figures are extensive and generally of high quality; however, as noted below, additional IF markers could be valuable. Overall, the results provide a fascinating glimpse into male meiotic X-chromosome dynamics that may ultimately reveal mechanisms of remodeling the chromosome axis.

Major Comments:

As the authors point out in the first sentence of the Discussion (line 339), this report is a phenotype description; the results do not yet provide mechanisms. A key issue yet to be resolved (and alluded to in lines 362-363) is whether the observed phenotypes are direct or indirect, perhaps even pleiotropic, effects of TRIM41 deficiency. While it is not necessary to prove this in such a first report on spermatogenic effects, the authors should caution against implicating specific mechanistic pathways. This is especially important because the phenotype is not a complete block of meiotic progression, which raises the issue of developmental and cell-specific timing of the effect(s) of TRIM41 deficiency. Of interest in this context, Fig. 2C reveals evidence for depleted number of spermatocytes in the KO, suggesting earlier effects of TRIM41 deficiency, perhaps on mitotic proliferation of spermatogonia. Another issue not fully addressed is the evidence that some KO spermatocytes undergo meiotic division and produce differentiating spermatids (Fig. 2C; but also Fig. 2A, where size of KO testis appears larger than in mutants with complete meiotic arrest). These issues and others could be more effectively addressed by providing information about the first wave of spermatogenesis in both mutant and control males. This strategy would allow the investigators to determine more precisely the developmental onset of aberrant phenotypes as well as to evaluate cell populations of comparable composition with respect to developmental stages, thereby diminishing concerns about secondary effects due to depletion of specific stages. The authors appear to make an attempt at this in determining frequency of stages, although Fig. 3D is hard to interpret and needs better legend and labeling. Overall, a developmental analysis would add valuable information and facilitate interpretation.

> We thank the reviewer for the valuable comment. Although we have examined spermatogenesis based on the seminiferous epithelium cycle, analyses of the first wave of spermatogenesis will be another important piece of information. We collected testes from juvenile male mice (Figs S3 and L. 173-176; PND8, PND12, PND16, and PND22). Consistent with the results of the adult testis, spermatogenesis defects started to appear from PND16, which correspond when the first wave of spermatogenesis reach pachytene.

L. 173-176: “Consistent with the results in adult testis, the weight decline of juvenile testis and apoptotic elimination of germ cells became prominent from PND16, corresponding to when the first wave of spermatogenesis reaches the pachytene stage (Figs S3A and S3B).”

The effects of TRIM41 deficiency on meiotic prophase are particularly striking. Overall, chromosome dynamics are impaired, but exactly how is not yet clear. Fig. 3B (but not Fig. 3F) suggests presence of univalents in the KO diplotene cells; this is an observation worth pursuing to determine the relative frequency of such cells.

- Thank you for the suggestion. As SYCP3 immunostaining (Fig. 3B) is difficult for us to determine the presence of univalents, we evaluated crossover defects by MLH1 immunostaining (Figs S12). In brief, MLH1 foci were also observed in SYCP3 overloaded regions. This result suggested that crossover-formation activity remains in *Trim41* KO spermatocytes.
- Considering this result, we removed the sentences regarding crossover formation defects from the manuscript.

The issue of SYCP3 "overloading" and its relationship to synaptic configurations (synapsis, un-synapsis, de-synapsis, and nonhomologous synapsis) is not yet clear. Additional IF makers, for example, HORMAD proteins, might provide more information.

- We tried HORMAD1 immunostaining and noticed that HORMAD1 remained on autosomes with overloaded SYCP3 (Fig. S9 and L.257-261). This result was consistent with BRCA1 signals on autosomes with overloaded SYCP3, further corroborating that autosomal synapsis was misregulated in *Trim41* KO spermatocytes.

L. 257-261: “Since HORMA domain-containing proteins localize to unsynapsed axes and function in the quality surveillance of homolog alignment and activation of the DNA damage response [31, 32], we examined HORMAD1 localization by immunostaining (Figs S9A and S9B). Consistent with BRCA1 immunostaining results, HORMAD1 remained on autosomes with overloaded SYCP3 (Fig. S9B; white arrowheads).”

The authors suggest that dense BRCA1 indicates meiotic silencing of unpaired chromatin (MSUC), but RNA FISH would be required for this conclusion.

- We agree with the necessity of RNA FISH for a clear conclusion. However, probe design against the (probably) random site on autosomes is quite difficult and beyond the scope of this revision. Therefore, to avoid misleading future research, we deleted the sentences regarding the meiotic silencing of unpaired chromatin (MSUC).

Importantly, SYCP3 "overloading" is accompanied by aberrations in deposition of SYCP3 and other proteins. The images of "overloading" are virtually all of a regular, rod-shaped, crystal-like structure totally atypical of the dense unpaired axes of the X chromosome, which can frequently exhibit dense staining for SYCP3 without assuming such a rigid configuration. This crystal-like structure should be further investigated, and the authors might find useful directions from recent studies showing liquid-crystal effects in assembly of the SC and the XY body (recent publications from the Dernburg and Namekawa labs would be valuable additions to both Introduction and Discussion sections).

- We thank the reviewer again for the valuable suggestion. Regarding XY body, we added Namekawa lab's recent review paper (Alavattam AG *et al*. Cell Mol Life Sci. 2021: PMID: 34971404.) to both Introduction and Discussion sections (L. 86-89 and L.382-384).

L.86-89: “However, unlike autosomes that fully synapse between homologs, sex chromosomes (X and Y in mammals) only synapse at the pseudoautosomal region (PAR) and are sequestered into a physically separated nuclear territory known as the XY body [13].”

L.382-384: “The XY body [13] malformation phenotype also indicated that *Trim41* KO spermatocytes failed in the physical separation of their sex chromosome.”

[13] Alavattam KG *et al.* Meiotic sex chromosome inactivation and the XY body: a phase separation hypothesis. Cellular and molecular life sciences : CMLS. 2021;79(1):18.

- Evaluation of the Liquid-crystal effects of SC will surely provide important information. We noticed that DNA staining was weak in the centerline of rod-shaped SYCP3, suggesting that some SC might not associate with DNA. We added the result to the manuscript (Fig. S5C and L. 203-206) and the previous research (Rog O *et al*. Elife. 2017. PMID: 28045371) showing crystal-like structure of the synaptonemal complex to the Discussion sections (L.414-416).

L. 203-206: “Of note, the centerline with intense SYCP3 signals showed weak γH2AX and DNA staining (Fig. S5C; white arrowheads), suggesting some fraction of the overloaded SYCP3 might not associate with chromosomes.”

L. 414-416: “Another possible mechanism of SYCP3 overloading in *Trim41* KO is that SYCP3 was misassembled into an alternative structure, which does not associate with chromosomes [45, 46]”

[45] Hughes SE, Hawley RS. Alternative Synaptonemal Complex Structures: Too Much of a Good Thing? Trends in genetics: TIG. 2020;36(11):833-44.

[46] Rog O, Köhler S, Dernburg AF. The synaptonemal complex has liquid crystalline properties and spatially regulates meiotic recombination factors. eLife. 2017;6.

Minor Comments:

All figures need review for clarity of labeling and legends.

Fig. 1J - clarify in label that it is female

Font of labels for proteins detected by IF should be same color as in image (e.g., red or green)

As mentioned above, Fig. 3D needs better explanation

Fig. 3H, x-axis - "chromosome" (not "chromsome")

- We appreciate the reviewer for clarifying the errors. We revised all the mistakes.

Reviewer #4 (Significance (Required)):

The value of this report lies in the new and fascinating implications of the aberrant accumulation and assembly of SYCP3 and other proteins of the SC on chromatin in the absence of TRIM41. Even though pathways and molecular mechanisms have not yet been revealed, this report will be of considerable interest to investigators of meiotic chromosomal dynamics (such as for example, this reviewer, who has expertise in genetics, meiosis and mutations affecting male fertility). The implications are that ubiquitin dynamics are essential for the underlying remodeling of chromosome axes that occurs in a temporally regulated manner during meiotic prophase. This phenotype description is a robust foundation that will underlie and propel future molecular analyses, as suggested by the authors in their conclusions.

**Referee Cross-commenting**

I think all of the reviewer comments are well-considered and cogent. Most, if followed by the authors, will improve the manuscript in a relatively short time frame. In my view, this is a report on an intriguing phenotype, not a mechanistic paper. As such, it deserves to be published in an appropriate journal in a timely manner because the forthcoming mechanistic studies are likely to take several years. (Reviewer 4)
