# Supplementary material for: Trim41 is required to regulate chromosome axis protein dynamics and meiosis in male mice
Source: PLoS Genet. 2022 Jun 1;18(6):e1010241. doi: 10.1371/journal.pgen.1010241 (PMC9191731; doi:10.1371/journal.pgen.1010241)
Supplement: Attachment — Submitted filename: Response to Reviewers.docx [file pgen.1010241.s023.docx]

**Comments to the Authors:
Please note here if the review is uploaded as an attachment.**

Reviewer #1: The authors corrected all my major comments on the previous version. The new data and edited text and figures are much improved.

Reviewer #2: In this manuscript, the authors analyse meiotic phenotypes of Trim41 knockout (KO) male mice and conclude that, surprisingly, this ubiquitin ligase E3 is essential for proper meiotic progression and fertility in male mice. The deletion of the RING domain phenocopy the KO mice suggesting that the E3 ubiquitin ligase activity is likely required for the meiotic functions. They show that spermatocytes of both mutant mice exhibit SYCP3 overloading in pachytene chromosomes, especially on the X chromosome. ΔRING-TRIM41 accumulated on the chromosome axes with overload SYCP3 which suggests a function on the chromosome axes. However they were unable to co-immunoprecipitate SYCP3 with TRIM41 or provide any direct molecular evidence of a direct interplay between them. The manuscript is merely descriptive and does not provide any mechanism to support their conclusions. Most if not all of the figures are wrongly numbered and ordered, which makes it difficult to read and review the paper. Plos Genetics is a journal of high quality and the articles that are submitted should be correctly edited to facilitate the work of the reviewers.

- We are sorry for the confusion caused by the wrong order of the figures. We revised all the mistakes.

In the present MS the authors used three mouse models: one KO, one transgenic, and a ΔRING and performed comprehensive phenotypic analyses. However, the strategy to develop the KO is not explained at all and they do not show that the targeting strategy actually gives rise to a null allele due to the fact that the antigen (residue 35-85 aa) used for the production of anti-TRIM41 antibodies is deleted in the KO (Fig S2C). A crucial point of the MS would be if there is or not a truncated protein being expressed despite the presence of the Neo cassette. The targeting construct employed also appears to lack the RING domain. Thus, it seems quoite feasible that the two mutant models (KO and ΔRING) are indeed equivalent / redundant. If so, it would not be necessary to present the same experiments in the 2 models. The authors must explain the strategy used in both models (show the region eliminated in the KO and why they consider that a null allele is generated and not a hypomorphic allele or a protein without E3 ligase activity; include the ssODN, etc…) in order to clarify this relevant question.

- To develop the KO, we replaced exon1 with Neo cassette as described in Fig 1I. The short and long arm of the targeting vector corresponds to chr11: 48,707,466–48,708,476 and chr11: 48,697,891– 48,706,384 (GRCm39), respectively. We added the explanation to the material & methods section (L. 441-444).
  **L. 441-444**: “To generate Trim41 KO mice, we replaced exon1 with a neomycin resistance (Neo) cassette in EGR-G101 ES cells [25] by transfecting a targeting vector. The short and long arm corresponds to chr11: 48,707,466–48,708,476 and chr11: 48,697,891– 48,706,384 (GRCm39), respectively.”
- As we could not raise an antibody against the C-terminal region, we instead performed RT-PCR using primers on exon2 and exon6. As the result, we did not find PCR amplicon in *Trim41*^neo/neo^ testis, although found in *Trim41^ΔRING/ΔRING^* counterparts. This result indicated *Trim41* expression was disturbed in *Trim41*^neo/neo^ mice. To clearly show the difference between the two mutant models (KO and ΔRING), we described the result in Fig. 6 and L.281-282.
  **L. 281-282**: “RT-PCR analysis confirmed the gene expression in *Trim41^ΔRING/ΔRING^* testis, albeit not in *Trim41^neo/neo^* counterparts (Fig. 6C).”

In lane 224-225 the authors affirm that “The result showed the X chromosomes with more frequent SYCP3 overloading than autosomes (Fig. 4C; X chromosome: 37 out 43 cells; autosome: 12 out 43 cells)”.That is 86% showed the X chromosome with SYCP3 overloading vs 28% with an autosome. However in Fig 3D, 148/207 cells (72%) shows Sycp3 overloading on both sex chromosomes and autosomes. These results are confusing; in the experiment of Fig 3D it appears that in most cells (72%) both sex chromosomes and autosomes are affected but in Fig 4C only 28% of the cells show affected the autosomes. Moreover, most if not all of the pictures of the KO cells show Sycp3 overloading on both type of chromosomes. This is incosnsitent.

- In mFISH experiments (Fig. 4), we might have missed minor abnormalities of SYCP3 signals such as those seen in the lower left and right of Fig. 3C because of harsh conditions of FISH procedure. We described this point in L. 227-230.
  **L.227-230**: “The result showed the X chromosomes with more frequent SYCP3 overloading than autosomes (Fig. 4C; X chromosome: 37 out 43 cells; autosome: 12 out 43 cells), although we might have missed minor abnormalities of SYCP3 signals because of harsh conditions of FISH procedure.”

In relation to Fig 3F, do the authors find any “normal” metaphase/anaphase?? What percentage are abnormal?

- We reanalyzed the immunostaining images (Fig. 3F) and found a small population of normal-looking spermatocytes. Regarding the SYCP3 staining, 96% of metaphase I and 97% of anaphase I spermatocytes exhibited rod-like SYCP3 signals. Chromosome mislocation was found in 11% of metaphase I and 17% of anaphase I spermatocytes. Also, in Giemsa staining images (Fig. 3G), 82% of metaphase I spermatocytes showed rod-like structured chromosomes. We described these results in L. 212-220.
  **L.212-220**: “The overloaded SYCP3 remained even in metaphase I and anaphase I (Fig. 3F; spermatocytes with rod-like SYCP3 signals: 0/308 [Het, metaphase I], 327/341 [KO, metaphase I], 0/50 [Het, anaphase I], 29/30 [KO, anaphase I]). In addition, some chromosomes connected with SYCP3 failed to align at the equatorial plate and were left behind between two centrosomes (Fig. 3F; white arrowheads; observed chromosome mislocation: 33/308 [Het, metaphase I], 2/341 [KO, metaphase I], 1/50 [Het, anaphase I], 5/30 [KO, anaphase I]). Probably because of the remaining SYCP3 axes, one or two chromosomes exhibited a rod-like structure at metaphase (Fig. 3G; black arrows; spermatocytes with rod-like chromosomes: 0/112 [Het]; 106/130 [KO]). These results showed that the SYCP3 dynamics on chromosome axes were misregulated in Trim41 KO spermatocytes.”

In Figure 5E and table S3 the number of late pachytene cells analysed per mouse is too low to obtain a conclusion (6, 10, 5, 4, 12 and 9 cells). Authors should increase the number of cells analysed.

-We stained additional samples with anti-SYCP1/SYCP3 antibodies (total: 5 males for each genotype) and revised Figure 5E, Table S3, and L. 250-254.
  **L.250-254**: “The immunostaining showed that SYCP1 on the sex chromosomes tended to remain in KO (Figs 5A, 5B, and 5E; SYCP1 remaining on the sex chromosome (Blue arrowheads): 190/359 [Het, early pachytene], 9/120 [Het, late pachytene], 206/254 [KO, early pachytene], 64/102 [KO, late pachytene]).”
  **Table S3 (SYCP3&SYCP1)**:

The numerical data of table S3 about the quantification of BRCA1 do not correspond to the text or to figure 5F. These data should be reviewed by the authors in order to clarify the conclusion reached.

- We apologize for the confusion again. “XY” in Table S3 showed the number of spermatocytes with even BRCA1 signals through their sex chromosome axes, which was the opposite way of presentation in Figure 5F. To avoid confusion, we changed the label in Table S3 to “uneven BRCA1 staining on sex chromosomes” and reversed the number (i.e. subtracted from the total number).

Reviewer #3: In this manuscript, resubmitted from Review Commons, the authors present information on the male infertility phenotype of mice lacking TRIM41, a ubiquitin ligase E3 protein. They report on creation of KO of theTrim41 gene, originally to investigate its role in innate immunity. Surprisingly, however, the KO males were infertile, with arrest of spermatogenesis in the meiotic stage. The synaptonemal complex protein SYCP3 exhibited “overloading,” especially on the unsynapsed X chromosome. Mice lacking the RING domain of TRIM41 phenocopied the KO, providing evidence that the ubiquitin ligase E3 activity is essential for normal accumulation of SYCP3 on chromosome axes and especially for the unsynapsed X-chromosome dynamics. These results are important and noteworthy in that they provide new information about male meiotic X-chromosome dynamics and may lead to further defining the mechanisms that remodel and develop the chromosome axis. Overall, this report is a very interesting phenotype description that lays the foundation for additional investigations and will attract considerable attention in the fields of meiosis and reproductive genetics.

As one of the previous reviewers, I find that the authors have provided a well-reasoned response to the previous review critiques and the manuscript has been appropriately revised to mitigate previous concerns and avoid over-reaching conclusions. The quality of the figures and imaging of SYCP3 labeling have been improved. Importantly, the authors have clarified that while the phenotype informs us about processes requiring TRIM41, it does not reveal precise mechanisms by which TRIM41 acts. For instance, we do not know how its localization is regulated or its substrates, nor do we yet know which aspects of the meiotic infertility phenotype are direct or indirect effects of the deficiency in TRIM41. Nonetheless, these limitations do not diminish enthusiasm for this nice phenotype description, which is an essential step toward unraveling mechanisms.

Overall, descriptions of strategies and data are clearly presented, analyses are appropriately controlled, and the figures are of high quality. However, in the compiled manuscript PDF provided for review, there were multiple errors (probably at the level of Editorial Manager) in assembly and labeling of figures. The labels on the authors’ figures are correct and figures match the legends; the headers inserted by Editorial Manager at the top of each figure page are frequently incorrect, leading to assembly of figures in the wrong order. This confusion must be resolved.

- We are sorry for the confusion caused by the wrong order of the figures. We revised all the mistakes.
